# Primary renal malignant epithelioid angiomyolipoma with distant metastasis: a case report and literature review

**DOI:** 10.3389/fonc.2023.1207536

**Published:** 2023-08-22

**Authors:** Jun Zhang, Wen-Juan Wang, Li-Hong Chen, Ning Wang, Ming-Wen Wang, Hao Liu, Li-Juan Pang, Han-Guo Jiang, Yan Qi

**Affiliations:** ^1^ Department of Pathology, Zhanjiang Central Hospital, Guangdong Medical University, Guangdong, China; ^2^ Department of Pathology, Shihezi University School of Medicine & the First Affiliated Hospital to Shihezi University School of Medicine, Xinjiang, China

**Keywords:** renal epithelioid angiomyolipoma, liver metastasis, histopathology, immunohistochemistry, differential diagnosis

## Abstract

Epithelioid angiomyolipoma (EAML) is a rare type of mesenchymal angiomyolipoma with potential malignancy in the kidney that can cause lymph node metastases, local recurrence, and distant metastases. Herein, we describe a case of EAML in the right kidney of a 51-year-old man who was admitted to the hospital with a right abdominal mass. Computed tomography revealed a heterogeneously enhanced mass with blurred margins, which was considered a malignant tumor. A radical nephrectomy was then performed. Two years later, the patient developed liver metastases from EAML and was administered sintilimab combined with bevacizumab. The patient survived after 6 months of follow-up. Histologically, the tumors showed clear boundaries and no obvious capsules. The tumor tissue mainly consisted of epithelioid tumor cells, thick-walled blood vessels, and a small amount of adipose tissue. Tumor cells with lipid vacuoles and acinar areas were large, round, polygonal, eosinophilic, or transparent in the cytoplasm. The enlarged and hyperchromatic nuclei were accompanied by distinct nucleoli and pathological mitosis. These histopathological findings resembled those of renal cell carcinoma, and immunohistochemical analysis was performed. The tumor cells were diffusely positive for HMB45, Melan-A, CK20, vimentin antibodies, and TFE3, suggesting that the tumor originated from perivascular epithelioid cells, excluding renal cell carcinoma. The Ki-67 index was 10%. These histopathological features were observed in liver mass puncture tissues. We also summarized 46 cases of EAML with distant metastasis and explored the clinicopathological features of EAML to improve the treatment of the disease. EAML is often ignored in the clinical setting, leading to metastasis and recurrence. Therefore, EAMLs require long-term follow-up, and timely detection of recurrent disease can improve the prognosis.

## Introduction

Renal angiomyolipoma (AML) belongs to the perivascular epithelioid tumor (PEComas) family of lesions, which originate in the mesenchymal tissue and are characterized by the coexpression of melanocytes (Melan) and muscle markers by perivascular clear and epithelioid cells (1). Epithelioid angiomyolipoma (EAML) is a rare and special subtype of AML. EAMLs have a major epithelioid component and potentially malignant behavior; they mainly consist of epithelioid cells with diverse morphology and abundant proliferation arranged in sheets, and the proportion of mature adipocytes tends to be <5% (2, 3). It can occur in the kidney, liver, bone, ileum, pelvic retroperitoneum, and most commonly in the kidney, with local invasion or metastasis. About one-third of patients may have lymph nodes, liver, lung, or spinal metastasis (4).

Recently, several cases of malignant EAML have been reported. Edmund et al. first reported a malignant EAML with liver metastasis in 2001 (5). However, only 17 studies have reported malignant EAML with liver metastasis at present (**Table 1**) (5–8, 11, 12, 16, 20–25, 27, 29, 32, 34). Additionally, there are no established criteria for predicting malignancy. The diagnosis of EAML may be challenging. In particular, the definite diagnosis of malignant EAML is established and confirmed by histological immunohistochemical findings owing to the similarity of its epithelioid morphology with that of renal cell carcinoma (RCC). Therefore, insights into the morphological characteristics and immunophenotype of this disease entity can aid in an accurate diagnosis.

**Table 1:** Clinical characteristics of the reported cases of EAML.

Case	Published time	Author		Sex/age	Site/location/size (cm)	Clinical symptoms and imaging features	TSC	Metastasis/time of occurrence (month(s))	Outcome and F-up (month(s))	IHC
1	2001	Edmund et al. (5)		F/49	Both/–/4*3*3	Recurrent urinary tract infections/B-ultrasound: hyperechoic masses	None	Liver/24	Live/28	HMB45, Melan-A
2	2001	Radin et al. (6)		M/21	Both/–/1–3.5	Gross hematuria/CT: irregular enhancing wall and central low attenuation	Yes	Liver, spleen, peritoneum, pleura retroperitoneal lymph nodes/5	–/–	HMB45, vimentin, NSE
3	2002	Takumni et al. (7)		M/47	L/U/20*10*10	Acute upper abdominal pain/CT: enhanced unequally mass	None	Lung, liver/2	Died/5	HMB45, SMA, EMA
4	2004	Warakaulle et al. (8)		F/48	L/I/15*14*11	A left-sided abdominal mass/ultrasound: hypoechoic mass	None	Liver/–	Live/10	HMB45, Melan-A, S100, CD10
5	2007	Huang et al. (9)		F/78	L/I/12.5*7.5*8.5	Fever and left flank pain/CT: a heterogeneous mass	None	Lung, bone, regional lymph node/4	Died/5	HMB45
6	2008	Moudoun et al. (10)		F/31	Both/–/1–10	Abdominal mass and intermittent left flank pain/–	Yes	Retroperitoneal lymph nodes/–	Live/12	HMB45, vimentin, NSE
7	2008	Sato et al. (11)		M/36	Both/–/20	–/–	Yes	Renal arterial wall infiltration, lung, liver, diaphragm, mesentery/24	Died/24	HMB45, Melan-A, vimentin, CD68, CD63, CD117
8	2011	Nese et al. (12)	1	F/24	–/–/–	–/–	Yes	Pelvic, liver/–	Died/12	–
			2	M/29	–/–/–	–/–	Yes	Lung, liver/18	Died/18	–
			3	M/14	–/–/11	–/–	Yes	Lymph node/–	Live/240	–
			4	F/49	–/–/34	–/–	Yes	Colon/–	–/–	–
			5	F/25	–/–/8	–/–	Yes	Lymph node, peritoneal, liver, lung/–	Died/12	–
			6	M/36	–/–/28	–/–	Yes	Lung, liver, mesentery, diaphragm/–	Autopsy/–	–
			7	M/67	–/–/15	–/–	None	Lymph node/–	–/–	–
			8	M/69	–/–/13	–/–	None	Liver, lymph node/8	Died/28	–
			9	F/46	–/–/17	–/–	None	Liver, peritoneum/12	Live/16	–
			10	M/36	–/–/29	–/–	Yes	Lung, liver/–	Died/4	–
			11	M/58	–/–/37	–/–	None	Lymph node, liver/–	Died/24	–
			12	M/27	–/–/11	–/–	None	Liver/–	Died/24	–
			13	M/29	–/–/27	–/–	Yes	Liver/–	Died/11	–
			14	F/55	–/–/12.8	–/–	None	Extensive metastatic disease/–	Died/12	–
			15	M/57	–/–/4.5	–/–	–	Lymph node/58	Live/58	–
9	2012	Lee et al. (13)		F/63	L/–/–	Left abdominal pain/–	None	Relapse in situ/3	Died/5	HMB45, Melan-A, SMA, vimentin, desmin
10	2012	Li et al. (14)		F/55	L/–/7.5	Left flank pain/ultrasonography: solid mass	None	Lung/84	Died/180	HMB45, P53
11	2013	Yang et al. (15)		F/42	R/–/–	–/B-ultrasound: substantial occupation	None	Lung/48	Live/55	HMB45, SMA, Melan-A
12	2013	Xi et al. (16)		M/7	R/U/15*12*8	Mild abdominal pain/CT: a heterogeneous mass	None	Lung, liver/6	Died/24	HMB45, Melan-A
13	2014	Shi et al. (17)		M/48	R/–/14*11*8	Abdominal pain and blood in urine/–	None	Lung/60 ileum/72	Live/148	HMB45, Melan-A, SMA, S100
14	2014	Wang et al. (18)		F/63	–/–/–	–/–	None	Lung/48	–/–	HMB45, vimentin, Melan-A, SMA
15	2014	Zhao et al. (19)		M/49	R/–/–	–/CT: masses of uniform density	None	Lung/36	Live/50	HMB45, Melan-A, SMA, desmin, S100, CD34
16	2014	Fukaya et al. (20)		F/22	R/–/21	–/–	None	Retroperitoneum, liver/84	Live/120	HMB45, SMA, E-cadherin, β-catenin
17	2015	Guo et al. (21)		F/48	R/I/13*12*11	Flank pain in the right-side/CT: a soft tissue mass of heterogeneous density	None	Lung, liver/16	Died/22	HMB45, Melan-A, desmin
18	2016	Xiao et al. (22)	1	–/–	L/–/–	–/CT: mass of heterogeneous density	None	Lung/–	Died/9	HMB45, Melan-A, CD117, SMA
			2	–/–	R/U/10	–/CT: mass of heterogeneous density	None	Liver/–	Died/21	HMB45, Melan-A, CD117, SMA
19	2016	Shen et al. (23)		M/36	L/–/–	–/–	None	Lung, liver/24	Live/5	HMB45, Melan-A, CD117, S-100, vimentin
20	2016	Park et al. (24)		M/48	–/–/–	–/–	None	Scapula, liver, pelvic bone, peritoneal seeding/12	Live/32	HMB45, Melan-A
21	2016	Cho et al. (25)		F/47	L/–/10.7*10*7.5	Acute left abdominal pain/CT: a well demarcated, heterogeneously enhancing, necrotic mass with renal vein thrombosis	None	Liver/1	Live/1	HMB45, vimentin, α-SMA, CD10
22	2018	Wang et al. (26)		F/53	L/–/11.9*10.0*10.1	Gross hematuria with presence of lumbago and fatigue/CT: ill-defined, irregular, slightly hyperdense mass	None	Lung/4	Died/10	HMB45, Melan-A, TFE3
23	2018	Zhan et al. (27)		F/48	R/–/7.5*6*4	–/CT: a well-defined solid tissue mass	None	Liver/13	Live/13	HMB45, Melan-A, SMA
24	2018	Park et al. (28)		F/36	L/–/10*13	Abdominal pain/–	None	Rectus abdominis muscle/60	Live/72	HMB45, Melan-A, CD117
25	2019	Bree et al. (29)		M/60	L/–/14*12*13	–/–	None	Spleen, liver, pelvis/84	Live/192	HMB45, Melan-A, SMA
26	2019	Erickson et al. (30)		F/–	–/–/–	Abdominal discomfort/–	None	Paraortic lymph node/–	–/–	HMB45, Melan-A
27	2020	Umair et al. (31)		F/31	Both/–/–	–/MRI: high signal intensity	None	Lung/–	Live/6	–
28	2020	Gupta et al. (32)		F/40	R/–/10.5*11.9*16	Abdominal pain/CT: heterogeneously enhancing mass	None	Liver/–	Died/2.5	Melan-A, SMA
29	2020	Fujiwara et al. (33)		M/37	R/–/11	Abdominal pain/–	None	Right-sided transverse colon/72	Live/198	HMB45, Melan-A
30	2022	Isaac et al. (34)		M/57	L/–/–	Headache and sinus congestion/–	Yes	Lung, liver/2	Died/9	HMB45, Melan-A, SMA, CK
31	2022	Present case		M/51	R/U/13*9	A palpable mass/ultrasound: solid mass; CT: heterogeneously enhancing mass	None	Liver/24	Live/26	HMB45, Melan-A, SMA, vimentin, CK20, TFE3

F, female; M, male; L, left; R, right; Up, upper pole; In, inferior pole; TSC, tuberous sclerosis complex; F-up, follow-up time; IHC, immunohistochemistry; HMB45, human melanoma black 45; Melan-A, melanoma antigen; SMA, smooth muscle actin; CK, cytokeratin; EMA, epithelial membrane antigen; CD, cluster of differentiation; NSE, neuron-specific enolase; TFE3, transcription factor enhancer 3; “–” not known.

## Case report

A 51-year-old man with a history of a painless mass in the right upper abdomen for 20 days presented to the Department of Urology at Zhanjiang Central Hospital, Guangdong Medical University (Guangdong, China). A physical examination revealed a painless mass in the upper right abdomen. A computed tomography (CT) examination revealed a 13 cm × 9.7 cm × 13.8 cm heterogeneously enhanced mass with a blurred boundary in the right renal parenchyma. A high-density calcification shadow is observed. Radical right nephrectomy was performed for renal malignancy. Subsequent histological examination showed that the right kidney was 11 cm × 7 cm × 4 cm in size, with a fat capsule on the surface; the cut surface was gray-red and dark-red. No definite mass was observed in the renal parenchyma. A 14 cm × 13 cm × 8 cm mass with no capsule was linked to the kidney capsule. The tumor showed expansive growth and did not invade the perirenal fat. The section of the mass was reddish-gray yellow and soft in texture, and most necrotic changes were observed.

Resected tumor specimens were fixed in 10% neutral-buffered formalin and processed for immunohistochemistry using a standard protocol. Paraffin-embedded blocks were sectioned at a thickness of 5 μm and stained with hematoxylin–eosin and various antibodies. The antibody clones, working dilutions, and commercial sources are listed in **Supplementary Table S1**.

**Figure 1:**
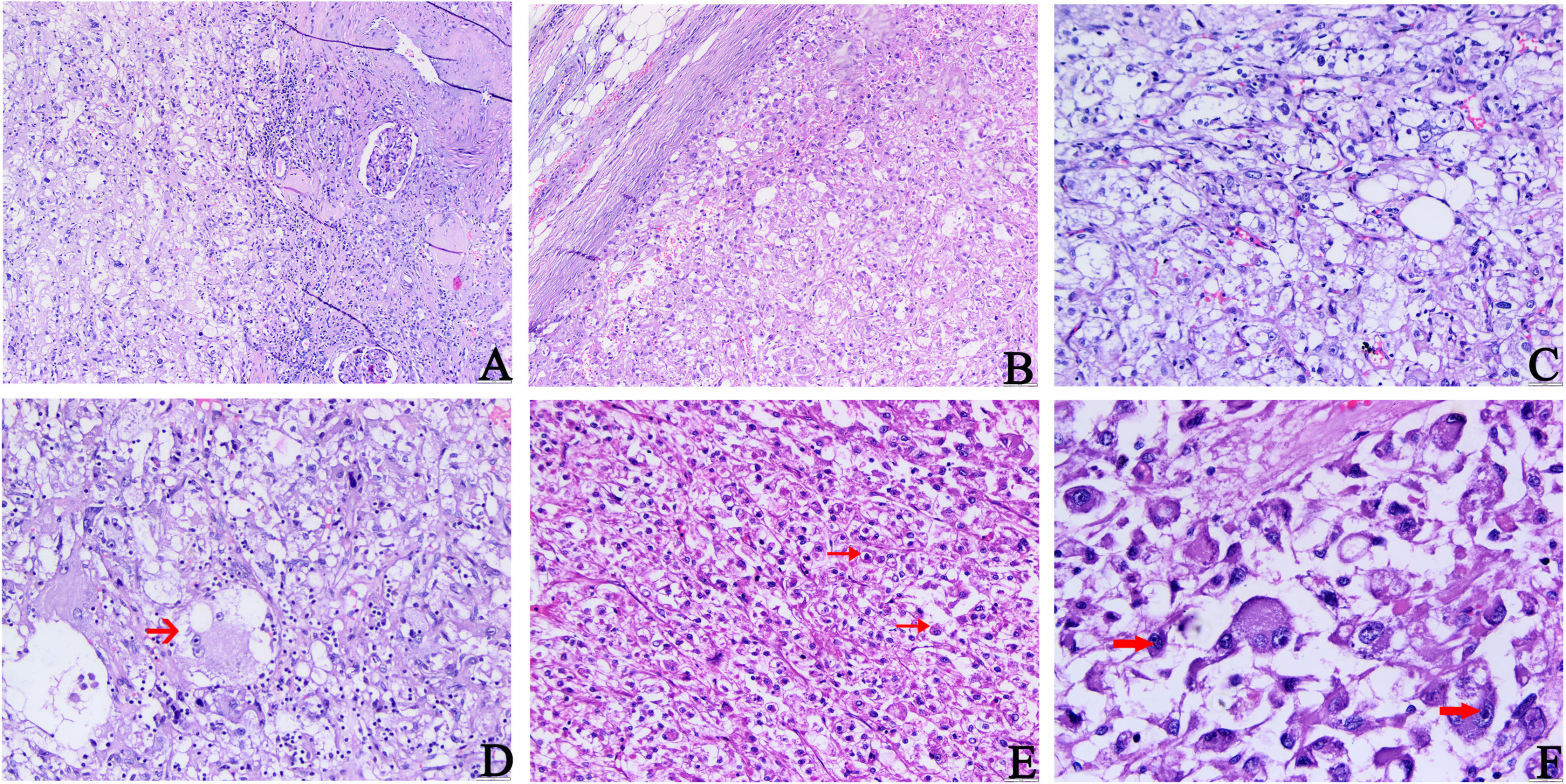
Histopathology features of primary tumors. **(A)** The boundary between tumor tissue and surrounding normal kidney tissue is not clear. **(B)** Epithelioid tumor cells were arranged in tight sheets. **(C)** Abundant epithelioid cells, a small amount of adipose tissue, and thick-walled blood vessels were seen in the tumor. **(D–F)** Epithelioid tumor cells are large and diverse, with some adipose vacuolar and acinar areas (indicated by the arrow in **(D)**), which are rich in eosinophilic or translucent cytoplasm. The nuclei were enlarged and deeply stained, partially vacuolated, and accompanied by obvious nucleoli (indicated by the arrow in **(F)**). The scattered megakaryocytes and occasionally pathological mitosis (indicated by the arrow in **(E)**) and necrosis could be observed (**A, B** H&E, ×100; **C–E** H&E, ×200; **F**, H&E, ×400).

Microscopically, there was no fibrous membrane around the tumor tissue, which was clearly demarcated from the surrounding normal renal tissue at low power. The tumor tissue consisted of numerous epithelioid cells, smooth muscle cells, twisted thick-walled blood vessels (hyalinized vascular walls), and small amounts of adipose tissue. The staining of tumor cells was shallower than that of normal renal cells. They were unevenly distributed. Thick-walled blood vessels, adipose tissue, and fiberglass lesions were observed in the cell-sparse areas. A large number of epithelioid cells were observed in the cell-rich areas. The epithelioid tumor cells were arranged in tight sheets (**Figures 1A–C**). They were large, round, or polygonal with some adipose vacuolar and acinar areas and had abundant cytoplasm, which was stained eosinophilic or transparent (**Figure 1D**). The enlarged nuclei were deeply stained with distinct nucleoli. Necrotic and pathological mitosis were also observed (**Figure 1E**). Vacuolated and weird-type nuclei were also observed (**Figure 1F**). These morphologies were easily confused with the histological features of RCC. Therefore, we performed an immunohistochemical examination.

**Figure 2:**
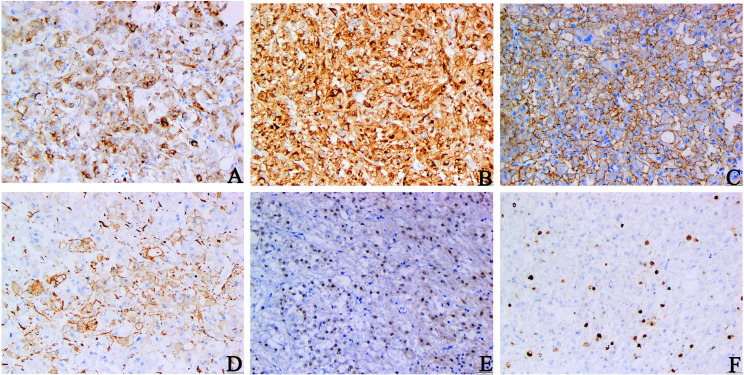
Immunohistochemical staining of primary tumors. **(A–C)** HMB45, Melan-A, CK20 staining: diffuse and strong positive cytoplasm of tumor cells. **(D)** SMA staining: tumor cells were positive. **(E)** TFE3 staining: tumor cell nuclear was positive. **(F)** Ki-67 staining: The Ki-67 index hit 10% (**A–D, F** original magnification is ×200; **E** original magnification is ×100).

Immunohistochemical staining revealed that the tumor cells were diffusively positive for human melanoma black 45 (HMB45), melan antigen (Melan-A), cytokeratin (CK) 20, and transcription factor enhancer 3 (TFE3), and partly positive for smooth muscle actin (SMA) (**Figures 2A–E**), which indicated that the tumor originated from peripheral vascular epithelioid cells, ruling out RCC. The tumor cells were diffusely vimentin-positive, and a Ki-67 index (**Figure 2F**) of 10% suggested that the tumor was malignant. The negative response of tumor cells to epithelial membrane antigen (EMA), PAX-8, CK, and CK7 excludes tumors of epithelial origin. The absence of melanocytes in the tumor tissue and the negative response of tumor cells to S100 and cluster of differentiation (CD) 117 precluded melanoma. Based on these pathological findings, the mass was confirmed as an EAML (**Supplementary Table S1**).

**Figure 3:**
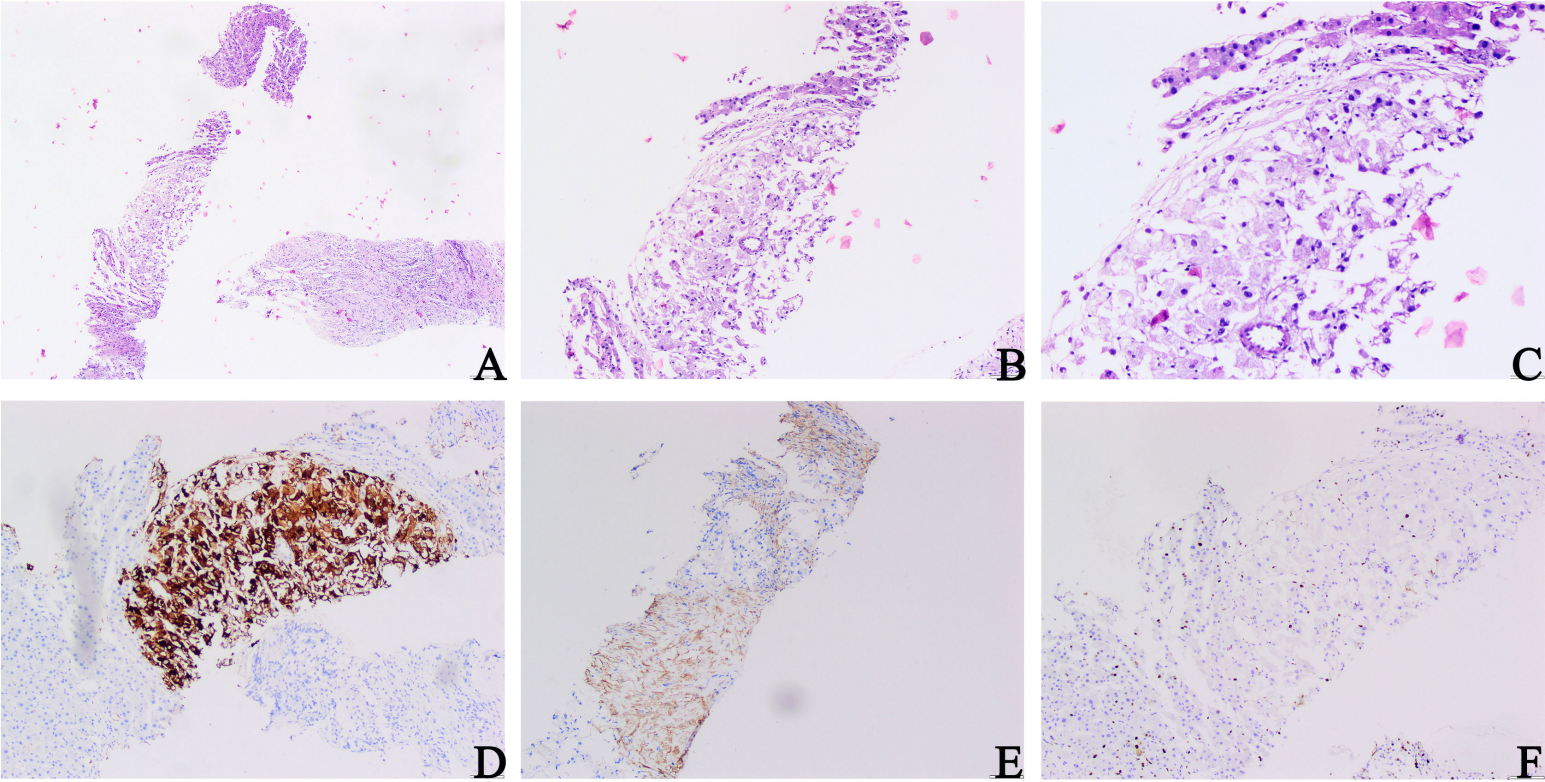
Microscopic architectural features of a liver mass biopsy. **(A–C)** Proliferating fibrous tissue, eosinophilic cytoplasm, and significantly hyperchromatic and enlarged nuclei of epithelioid tumor cells could be observed (H&E, **A** ×40; **B** ×100; **C** ×200). **(D)** Melan-A staining: diffuse and strong positive cytoplasm of tumor cells. **(E)** SMA staining: tumor cells were positive. **(F)** Ki-67 staining: the Ki-67 index hit 5% (**D, F** original magnification is ×200; **E** original magnification is ×100).

The patient was not treated or followed up after surgery. Two years later, the patient was admitted to the hospital with right lumbago pain, chills, and a fever for more than 1 week. A physical examination revealed tenderness in the right lumbar region without pain. CT examination showed multiple nodules with unequal density in the right nephrectomy area and a mass of 13.9 cm × 12 cm × 12.6 cm mixed with a slightly low-density shadow in the right lobe of the liver with unclear boundary and heterogeneous enhancement, which was considered a metastatic tumor. A biopsy of a liver mass was carried out for a pathological examination. Microscopically, only a small number of tumor cells were observed in the liver biopsy samples. Significant atypical epithelioid tumor cells with hyperchromatic nuclei were observed. The morphological characteristics were similar to those of the primary lesion (**Figures 3A–C**). Combined with the history of malignant EAML and imaging findings, we suspected that the patient had developed liver metastases. To confirm this hypothesis, we performed an immunohistochemical examination, which suggested diffuse positivity of liver tumor cells for Melan-A (**Figure 3D**) and SMA (**Figure 3E**). Its proliferation index hit 5% (**Figure 3F**). These findings confirmed the diagnosis of EAML metastasis. The patient or his family members had no history of tuberous sclerosis (TSC) or other renal tumors. The patient received sintilimab combined with bevacizumab. The patient survived after 6 months of follow-up.

## Discussion

AML is a renal tumor that accounts for 2%–6.4% of all renal tumors (2, 3). Renal EAML, a subtype of AML, is a rare renal mesenchymal tumor with malignant potential that was first reported by Mai et al. (35). The development of renal AMLs may be associated with TSC. It is a systemic autosomal-dominant disease characterized by hamartomas of the lungs, skin, heart, brain, and kidney (36). It is usually caused by reduced or missing expression of the TSC1 (hamartin) or TSC2 (tuberin) genes (37). Renal AMLs are found in 80% of patients with TSC (38). Similarly, analysis of sporadic AMLs and EAMLs showed an association with TSC2 (9, 38, 39).

To date, only 46 cases of EAML with distant metastasis have been reported in the literature (**Table 1**) (5–34). Through a review of the literature, we found that these tumors occurred in people over 40 years of age (26/43, 60%, ranging from 7 to 78 years old; median, 49 years; mean, 44 years), and there was no significant difference in sex. The patients were mainly affected by unilateral kidney disease (21/28, 75%), with the left side being the most affected (12/19, 63%). The size of the EAML tumors excised from the kidneys ranged from 1 cm to 37 cm, with an average size of 14.89 cm. A total of 12 patients (12/46, 26%) had TSC. The most distant metastatic sites were the liver (28/46, 60%), lungs (19/46, 41%), and lymph nodes (10/46, 21%). Metastases to other sites, such as the pelvis, peritoneum, and rectus abdominis, are less common (**Table 2**). Some patients had signs of distant metastasis before the primary tumor was found, and the time range of distant metastasis ranged from 1 month to 12 years after primary tumor resection in most patients, with an average of 2.7 years. The longest postoperative survival time was 20 years (12), and the shortest was 2.5 months (32). We found that the efficacy of surgery or chemotherapy after EAML metastasis was unsatisfactory, with a poor prognosis and a low 5-year survival rate.

**Table 2:** Sites of EAML distant metastasis with their percentages in the literature.

Metastasis sites	Case number (total: 46)	Percentage
Liver (5–8, 11, 12, 16, 20–25, 27, 29, 32, 34)	28	60%
Lung (7, 9, 11, 12, 14–19, 21–23, 26, 31, 34)	19	41%
Lymph node (6, 9, 10, 12, 30)	10	21%
Pelvic (12, 24, 29)	3	7%
Peritoneum (6, 12, 20)	3	7%
Spleen (6, 29)	2	4%
Retroperitoneal cavity (6, 10)	2	4%
Mesentery (11, 12)	2	4%
Relapse *in situ* (13)	1	2%
Rectus abdominis muscle (28)	1	2%
Ileum (17)	1	2%
Scapula (24)	1	2%
Colon (12)	1	2%
Bone (9)	1	2%
Extensive metastatic (12)	1	2%

Most patients present with abdominal pain, hematuria, palpable masses, or clinical symptoms of EAML that metastasize to the lungs and cause fever, cough, and chest pain. A CT examination is of great significance in planning surgery and predicting patient prognosis, as it may help discover the location of tumors and determine whether they have metastasized. EAML tumors were large (usually >7 cm), and CT showed irregular mixed-density solid or multilocular mass shadows (usually >45 HU) with uneven enhancement and “fast in and slow out.” This phenomenon may be associated with higher cell density, reduced tumor stroma, abnormal hyperangiogenesis, the presence of intact tumor capsules, and the absence of tissue structures with reflux vessels (3). The imaging findings of the mass in our case showed an inhomogeneously enhanced mass, which was considered to be a malignant renal tumor, consistent with the above summary of imaging findings. However, the above results may be misdiagnosed as RCC or retroperitoneal sarcoma (40, 41). Therefore, further pathological examinations are required to confirm the diagnosis.

In addition to mature adipocytes, smooth muscle-like spindle cells, and clear, thick-walled blood vessels, EAML contains a variety of clear-to-eosinophilic and cytoplasmic epithelioid cells. There is no uniform standard for determining the number of epithelioid cells required for a final diagnosis of EAML. Current inclusion criteria range from 10% to 95%, whereas the new edition of the World Health Organization in 2016 recommends greater than 80% epithelioid cells as the diagnostic criteria for EAML (42). A precise diagnostic criterion is beneficial for identifying the characteristics of EAML as it can be used as a guide for clinical treatment. Additionally, the diagnosis of malignant renal EAML is controversial, and there are no unified malignant diagnostic criteria. With the accumulation of clinical cases in recent years, researchers have found that recurrence and metastasis rates of the disease are as high as 17% and 49%, respectively, and the mortality rate can reach 33% (43). Therefore, highly invasive biological behaviors and histological features should initially be considered malignant.

Lei (44) concluded that three or more of the following characteristics predicted an increased likelihood of malignancy: (1) necrosis, (2) tumor size >9 cm, (3) tumor thrombus formation in the vein, and (4) epithelioid cells >70% or atypical cells >60%. These criteria greatly aid in the accurate diagnosis of malignant EAML. The proportion of tumor epithelioid cells in our case was 80%, which was consistent with the diagnosis of EAML. The tumor cells showed obvious atypia and hyperchromatic nuclei, accompanied by evident nucleoli, pathological mitosis, and necrosis, consistent with the diagnosis of malignant EAML. Two years later, the patient developed EAML liver metastases, which further confirmed the diagnosis of a malignant tumor. However, when tumor nuclear atypia is evident and the adipose tissue content is significantly reduced, these tumors are most likely to be misdiagnosed as RCC or sarcoma. Therefore, the final diagnosis depends on immunohistochemical staining of the tumor. EAMLs exhibit specific immunohistochemical characteristics. The tumor cells are positive for melanoma-related markers such as HMB45, HMB50, Melan-A, SOX10, and myogenic markers such as SMA but negative for epithelial markers such as AE1/AE3 and EMA (45, 46). Compared with EAML, epithelial markers for RCC were positive, and melanocyte markers were negative. While EAML epithelial cell marker staining was negative, HMB45 and Melan-A staining were generally positive. These cells also expressed SMA. Staining for S-100 protein is usually negative. In this case, the tumor cells were diffuse, strongly positive for HMB45 and Melan-A, and negative for EMA, CK, CK7, and PAX-8, excluding the diagnosis of RCC (**Supplementary Table S2**) (47–50). Moreover, melanocytes in the tumor tissue and their negative responses to S100 and CD117 excluded the diagnosis of melanoma. To our surprise, our case was strongly positive for CK20 staining, which had not been seen in previous case reports. We reviewed relevant reports on the expression of CK20 in epithelioid tumors and found that epithelioid malignant mesothelioma (51) and malignant hepatic epithelioid hemangioendothelioma (52) could abnormally express CK20. Perhaps our case could serve as the first report of an anomalous EAML expression of CK20. Interestingly, the positive expression of TFE3 in our case can help clinicians adjust the follow-up visit treatment strategy.

Currently, the treatment options for EAML include surgery, chemotherapy, targeted therapy, endocrine therapy, and immunotherapy. Surgical excision is the primary treatment of choice for renal EAML (53). However, some patients experience recurrence or even distant metastasis after surgery. Malignant EAML is prone to tumor thrombus formation, which may cause distant metastasis. A study found that mutations in TSC1/TSC2 and translocations in TFE3 lead to overactivation of the mTOR complex (54). MTOR inhibitors inhibit mTOR activity to control tumors. However, there have been cases of limited efficacy of mTOR inhibitors in TFE3-rearranged malignant PEComas, and targeting VEGF/VEGFR signaling is probably a new effective treatment strategy for TFE3-associated malignant PEComas (54, 55). Therefore, it is important to determine whether TFE3 is positive to guide subsequent treatments. Lattanzi et al. first reported a case of malignant EAML with a TSC mutation and resistance to mTOR inhibitor therapy. After switching to PD-1 antibody therapy, the patient’s disease was effectively controlled, suggesting that PD-1 antibody therapy is a breakthrough in the treatment of anti-malignant EAML. Therefore, the standard treatment for EAML with distant metastases is mTOR inhibitor-targeted therapy (everolimus) combined with PD1 immunotherapy (bevacizumab), which is the most effective treatment strategy in the current study (56). However, its therapeutic effects on EAML relapse and distant metastasis remain unsatisfactory. As in our case, the clinicians and patients did not pay attention to long-term return visits and active treatment, which resulted in liver metastases. Therefore, long-term follow-up visits and active treatment are of great significance for detecting recurrence or metastasis as early as possible in EAML. Since there was no mTOR inhibitor available in our hospital, our patient was treated with bevacizumab (200 mg dL^−1^) combined with sintilimab (200 mg dL^−1^), and the patient and his family agreed to this treatment strategy. The treatment was effective, and the patient was stable.

## Conclusion

EAML is a tumor of interstitial origin that expresses both myogenic and melanin markers. There is increasing evidence that it has malignant potential. Therefore, it is necessary to consider EAML other than RCC when dealing with intravascular thrombosis in renal tumors. The diagnosis is usually made based on histopathological examination; however, it is easily confused with other tumors, especially RCC. Immunohistochemical markers can be used for efficient differentiation to obtain an accurate diagnosis. Because of the risk of disease recurrence, which may occur very late, renal EAMLs require long-term follow-up to detect recurrence and metastasis as early as possible. In such cases, more efficient active treatment can be performed.

## Data availability statement

The original contributions presented in the study are included in the article/**Supplementary Material**. Further inquiries can be directed to the corresponding author.

## Ethics statement

The studies involving human participants were reviewed and approved by Zhanjiang Central Hospital, Guangdong Medical University. The patients/participants provided their written informed consent to participate in this study. Written informed consent was obtained from the individual(s) for the publication of any potentially identifiable images or data included in this article.

## Author contributions

JZ, M-WW and HL performed the experiments and analyzed the data; YQ designed and supervised the study. L-JP, H-GJ, NW and L-HC provided crucial input for the project; JZ, W-JW and YQ wrote the manuscript. All authors read and approved the final version of the manuscript.
