# Supplementary material for: Primary renal malignant epithelioid angiomyolipoma with distant metastasis: a case report and literature review
Source: Front Oncol. 2023 Aug 22;13:1207536. doi: 10.3389/fonc.2023.1207536 (PMC10477911; doi:10.3389/fonc.2023.1207536)
Supplement: Supplementary file 1 [file Table_1.docx]

| Primary Antibody | Clone | Source | Dilution | IHC finding |
| --- | --- | --- | --- | --- |
| HMB45 | HMB45 | Mouse | 1：80 | ++ |
| Melan-A | A103 | Mouse | Ready-to-use | ++ |
| CK20 | KS20.8 | Mouse | 1：150 | ++ |
| TFE3 | MRQ-37 | Rabbit | Ready-to-use | ++ |
| Vimentin | MX034 | Mouse | 1：120 | ++ |
| SMA | 1A4 | Mouse | 1：70 | + |
| CK7 | OV-TL 12/30 | Mouse | 1：100 | - |
| CK | AE1/AE3 | Mouse | 1：100 | - |
| RCC | PN-15 | Mouse | Ready-to-use | +/- |
| CD117 | YR145 | Rabbit | Ready-to-use | - |
| S100 | 4C4.9 | Mouse | 1：130 | - |
| EMA | E29 | Mouse | Ready-to-use | - |
| Ki-67 | MXR002 | Rabbit | 1：180 | 10%+ |
| D2-40 | D2-40 | Mouse | Ready-to-use | - |
| PAX-8 | MXR013 | Mouse | 1：100 | - |

**Supplementary Table 1. Primary Antibodies Used for Immunohistochemistry of EAML in this case.**

**Abbreviations:** HMB45, Human melanoma black 45; Melan-A, Melanoma Antigen; CK, Cytokeratin; TFE3, Transcription factor enhancers 3; RCC, Renal Cell Carcinoma; CD117, cluster of differentiation 117; EMA, epithelial membrane antigen. ++, Diffuse positive; +, Partly positive; -, Negative.
