# Supplementary material for: Primary renal malignant epithelioid angiomyolipoma with distant metastasis: a case report and literature review
Source: Front Oncol. 2023 Aug 22;13:1207536. doi: 10.3389/fonc.2023.1207536 (PMC10477911; doi:10.3389/fonc.2023.1207536)
Supplement: Supplementary file 2 [file Table_2.docx]

**Supplementary Table 2. Difference of clinicopathological data of EAML and RCC.**

|  |  | **EAML** | **RCC** |
| --- | --- | --- | --- |
| Age(years old) |  | 45-55(49) | 40-55(50) |
| Sex(male:female) |  | 1：4(49) | 2：1(50) |
| Pathological manifestations |  | It consists of cuff-like epithelioid cells, a back wall devoid of elastic membranes that twist blood vessels, smooth muscle tissue, and a small amount of adipose tissue (51). | Cubic tumor cells are arranged in a nest cord, the cytoplasm is transparent or eosinophilic, and the nucleus is round (52). |
| IHC findings(46-48) | HMB 45 | + | - |
|  | Melan-A | + | - |
|  | SOX-10 | + | - |
|  | CD117 | + | - |
|  | SMA | + | - |
|  | S-100 | + | - |
|  | EMA | - | + |
|  | TFE3 | +/- | - |
|  | vimentin | + | + |
|  | EMA | - | + |
|  | CK | - | + |
|  | RCC | - | + |
|  | CD 10 | - | + |
|  | AE1/AE3 | - | + |
|  | CK8 | - | + |
|  | PAX-8 | - | + |
| Clinical syndrome |  | TSC(26) | Von Hippel-Lindau Syndrome(50) |

**Abbreviations:**TSC, tuberous sclerosis complex; IHC, immunohistochemistry; HMB, human melanoma black; Melan-A, melanoma antigen; SMA, smooth muscle actin; CK, cytokeratin; EMA, epithelial membrane antigen; CD, cluster of differentiation; NSE, neuron-specific enolase; TFE3, transcription factor enhancer 3; SOX-10, Anti Human SOX-10.
